# Ultrathin bronchoscopy for solitary pulmonary lesions in a region endemic for tuberculosis: a randomised pilot trial

**DOI:** 10.1186/s12890-016-0225-1

**Published:** 2016-04-27

**Authors:** Daniel Franzen, Andreas H. Diacon, Lutz Freitag, Pawel T. Schubert, Colleen A. Wright, Macé M. Schuurmans

**Affiliations:** Division of Pulmonology, University Hospital Zurich, University of Zurich, Raemistrasse 100, Zurich, 8091 Switzerland; Department of Internal Medicine, Lung Unit, Tygerberg Academic Hospital, University of Stellenbosch, Cape Town, South Africa; Division of Anatomical Pathology, Tygerberg Academic Hospital, University of Stellenbosch, Cape Town, South Africa; NHLS, Port Elizabeth and Tygerberg Hospital, Cape Town, South Africa

**Keywords:** Solitary pulmonary nodule, Ultrathin bronchoscopy, Lung cancer, Diagnostic yield, Histology

## Abstract

**Background:**

The evaluation of solitary pulmonary lesions (SPL) requires a balance between procedure-related morbidity and diagnostic yield, particularly in areas where tuberculosis (TB) is endemic. Data on ultrathin bronchoscopy (UB) for this purpose is limited. To evaluate feasibility and safety of UB compared to SB for diagnosis of SPL in a TB endemic region.

**Methods:**

In this prospective randomised trial we compared diagnostic yield and adverse events of UB with standard-size bronchoscopy (SB), both combined with fluoroscopy, in a cohort of patients with SPL located beyond the visible range of SB.

**Results:**

We included 40 patients (mean age 55.2 years, 45 % male) with malignant SPL (*n* = 16; 40 %), tuberculous SPL (*n* = 11; 27.5 %) and other benign SPL (*n* = 13; 32.5 %). Mean procedure time in UB and SB was 30.6 and 26.0 min, respectively (*p* = 0.15). By trend, adverse events were recorded more often with UB than with SB (30.0 vs. 5.0 %, *p* = 0.091), including extensive coughing (*n* = 2), blocked working channel (*n* = 2), and arterial hypertension requiring therapeutic intervention (*n* = 1), all with UB. The overall diagnostic yield of UB compared to SB was 55.0 % vs. 80.0 %, respectively (*p* = 0.18). Sensitivity for the diagnosis of malignancy of UB and SB was 50.0 % and 62.5 %, respectively (*p* = 0.95).

**Conclusion:**

UB is not superior to SB for the evaluation of SPL in a region endemic with tuberculosis, when combined with fluoroscopic guidance only.

**Trial registration:**

ClinicalTrials.gov (Identifier: NCT02490059).

**Electronic supplementary material:**

The online version of this article (doi:10.1186/s12890-016-0225-1) contains supplementary material, which is available to authorized users.

## Background

Solitary pulmonary lesions (SPL) are frequently detected in computed tomography (CT) scans of the chest. A fourth of the patients who underwent a low-dose chest CT scan in the U.S. National Lung Screening Trial (NSLT) had a positive screening result; mostly presenting as one or more SPL or a pulmonary mass suspicious for lung cancer [1]. Thus, by following a lung cancer screening program with low-dose chest CT, an increasing number of SPL for evaluation is to be expected. However, more than 95 % of SPL detected by low-dose CT screening show false positive findings due to a non-malignant final diagnosis [1]. In a tuberculosis (TB) endemic area, the prevalence of benign SPL may be even higher [2], and therefore efforts have been undertaken to establish diagnostic modalities to distinguish between malignant and benign lesions without excess perioperative mortality [3]. According to guidelines for the diagnosis of SPL, there are three possibilities to manage these patients depending on the probability of an underlying malignancy: serial CT scans, tissue biopsy, or direct surgical resection [4]. Direct surgical resection is not justified in the majority of cases given the high rate of false positive findings in the context of SPL in a high TB burden environment. The goal is to diagnose malignant SPL promptly, to permit timely surgical resection, while avoiding invasive testing or surgery in patients with benign SPL [5]. The sensitivity of traditional transbronchial biopsy (TBB) with flexible bronchoscopy ranges between 14 % and 63 %, depending on size and location of the SPL [6]. Recently, new navigational bronchoscopy techniques have emerged which guide the bronchoscopist to the SPL using various image-processing modalities. These technologies including electromagnetic navigation bronchoscopy (ENB), virtual bronchoscopy (VB), and radial endobronchial ultrasound (R-EBUS) have shown to have a better diagnostic yield than traditional TBB [5]. In countries with limited resources, these technologies may not be available due to financial constraints.

Data on the utility of ultrathin bronchoscopy (UB) for the diagnosis of SPL is limited [7]. The smaller outer diameter of the instrument might improve access to SPL not visible by standard-size bronchoscopy (SB) and thus improve the diagnostic yield compared to TBB using SB. Based on the considerably smaller outer diameter of the UB it may be possible to visualise endoscopically more peripheral lesions and thus increase the yield of sampling in the area of interest. We undertook this prospective randomised trial to compare the overall diagnostic yield and adverse event rate of UB with that of SB for the diagnosis of SPL in lesions that were not visible on SB in a TB endemic region.

## Methods

### Study design and patient selection

**Fig. 1:**
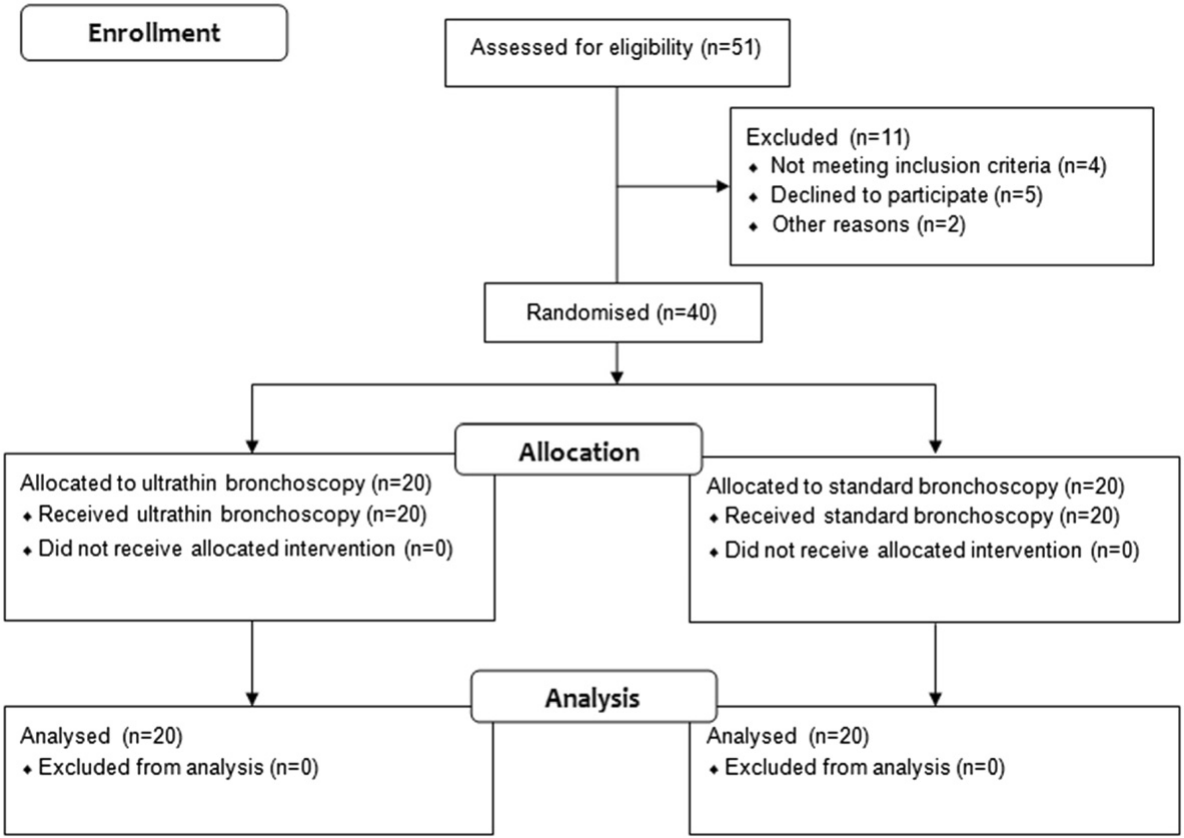
Study flow diagram (CONSORT flow chart)

The present prospective single-centre randomised pilot study was performed at Tygerberg Academic Hospital, a tertiary university hospital in Cape Town, South Africa, with a referral drainage area of 1.5 million people and TB notification rate of up to 1’000/100’000 persons per year when the study was performed. Between November 2000 and November 2003 all patients referred to the lung unit with single pulmonary lesion ≤ 6 cm in diameter on chest CT were considered. SPL was defined as a single and circumscribed pulmonary lesion with a diameter ≤ 6.0 cm, surrounded by aerated lung tissue, without evidence of satellite lesions, adenopathy or characteristic signs of malignant or benign lesions [3]. Location and maximal diameter of all indeterminate SPLs were recorded from the chest CT prior to enrolment of patients. Inclusion criteria were a previous cytological and microbiological negative sputum examination, absence of enlarged mediastinal or hilar lymph nodes on chest CT scan, and informed consent obtained before start of the procedure. Exclusion criteria were SPL with lesion size unchanged over two years, inability to undergo bronchoscopy or thoracotomy, and pregnancy. The study flow diagram is displayed in Fig. 1. Participants with consent for participation in the study in whom the lesion was found to be visible on SB were then not randomised and not considered part of the study population. Written informed consent was obtained from all patients before inclusion in the study, which was approved by the Committee for Human Research of Stellenbosch University (Cape Town, South Africa) with the reference number 2000/C094. The study is registered at ClinicalTrials.gov (Identifier: NCT02490059).

### Randomisation during bronchoscopic procedure

All procedures were started using SB with an external diameter of 5.0–6.0 mm with a biopsy channel of 2.2–2.8 mm (models Olympus BF-30 and BF-1 T160, Olympus, Tokyo, Japan). If during SB the lesion was endoscopically visible the bronchoscopy was continued as standard diagnostic procedure and the patients were excluded from the analysis. Only if no tumour was visible during complete inspection of the bronchial tree using the SB, a participant was randomised by opening a numbered sealed opaque envelope. Randomisation was performed using sequentially numbered (1–40) sealed opaque envelopes (block randomisation: block size 4). For subjects allocated to the SB group, the examination was immediately continued with the same SB bronchoscope. For subjects randomised to UB, the instrument was changed immediately to an Olympus BF-XP 40 ultrathin bronchoscope with an outer diameter of 2.8 mm and a working channel 1.2 mm during the same bronchoscopy session.

### Procedural details

Five experienced operators performed all procedures under topical anaesthesia (1 % lidocaine) and conscious sedation (intravenous midazolam). Sampling of SPL was performed with brush and forceps biopsies under fluoroscopic guidance, and with bronchial washing as deemed appropriate by the bronchoscopist. Using all modalities was encouraged by the protocol. Three or more samples were taken using the biopsy forceps and at least 2 samples using the brush. A study-specific case report form including a checklist on the bronchoscopic procedures and findings was completed by the bronchoscopist directly after the procedure. The lesion was considered “reached” if the biopsy forceps and/or brush were placed within the lesion, and it was considered “moved” when push and pull manoeuvers with the biopsy forceps and/or brush were transferrable to the lesion, both as assessed by two-dimensional fluoroscopic visualisation. Procedure duration time was measured from insertion of bronchoscope to extraction and in the case of randomisation to UB included the change over time from SB to UB. All laboratory staff was blinded to study group allocation. Biopsy specimens obtained by bronchoscopy were compared with the diagnosis made by surgical resection or CT-guided trans-thoracic needle aspiration if applicable. Efforts were made to obtain confirmatory histological diagnosis for all patients. Alternatively, when invasive diagnosis was not feasible or appropriate (as assessed by a multidisciplinary tumour board) radiological follow-up examinations were performed over two years.

### Outcomes

The primary outcome was to determine and compare the overall diagnostic yield and the sensitivity for the diagnosis of malignancy of UB versus SB. The diagnostic yield was calculated by identifying cases for which a definitive histological or cytological diagnosis could be made or follow-up examinations after two years suggested a benign disease. The following formula was used to compute the overall diagnostic yield: diagnostic yield (%) = 100 × cases diagnosed by UB or SB/total number of patients with completed procedures. The sensitivity was calculated as previously described [8], and is related to the diagnosis of malignancy. The secondary outcome was to compare the adverse event rate and procedure duration of UB versus SB.

### Adverse events

The intensity levels of the adverse events (AEs) were graded according to a standard three-grade scale [9]. Grade 1, or mild AE, was defined as “no effect on bronchoscopic procedure”; Grade 2, or moderate AE, was considered when “some interference with bronchoscopic procedure” occurred; and Grade 3, or severe AE, was present if “early termination of bronchoscopy was required”. For purpose of this study only grade 2 and 3 AEs were recorded.

### Statistical analysis

All statistical analyses were performed using IBM SPSS Statistics for Windows, version 22 (IBM corporation, Armonk NY, USA). Data are reported as median (interquartile range, IQR), or mean ± standard deviation (SD), or percentages as appropriate. Distribution of normality was tested with the one-sample Kolmogorov-Smirnov test. The difference between patients between the groups was calculated using the Fisher’s exact test (for 2 × 2 tables) or χ^2^ test for categorical data, and Mann–Whitney *U*-test or student’s *t*-test for continuous data. Both overall diagnostic yield and sensitivity between the groups were compared using the χ^2^ test*. P*-values of all outcomes were two-sided; a value less than 0.05 was considered to indicate statistical significance. There was no sample size calculation as the study was designed as a pilot trial to test feasibility.

## Results

Forty patients were included in the study (see Additional file 1). Baseline characteristics and the final diagnoses are displayed in Table 1. Neither localization of SPL in the upper lobes (*p* = 0.33), nor lesion size > 3 cm was associated with active TB (*p* = 0.21). Although none of the lesions were visible by SB (inclusion criterion), two of the 20 lesions (10 %) were visualized endoscopically by UB. The mean procedure time for UB and SB allocated patients was 30.6 and 26.0 min, respectively (*p* = 0.15). By trend, AEs were significantly more frequent in the UB compared to the SB group (30.0 vs. 5.0 %, *p* = 0.091). AEs in the UB group included extensive coughing (*n* = 2), blocked working channel or weak suctioning (*n* = 3), and hypertensive urgency requiring therapeutic intervention (*n* = 1). No grade 3 AEs were documented. In the SB group, there was one case of extensive bleeding, which was stopped by topical application of adrenaline through the working channel of the bronchoscope. Procedural details were equally distributed within the groups (Table 2). The overall diagnostic yield of fluoroscopic guided UB compared to SB was 55.0 % vs. 80.0 %, respectively (*p* = 0.18). Sensitivity for the diagnosis of malignancy of UB and SB was 50.0 % and 62.5 %, respectively (Table 3). Rates of true positives, true negatives and false negatives in UB compared to SB were 20 % vs. 25 %, 60 % vs. 60 %, and 20 % vs. 15 %, respectively. The diagnostic yield of lesions that were fluoroscopically moved was 90.9 % compared to 88.0 % in lesions that were fluoroscopically reached. However, only a “reached” SPL was a significant predictor of the diagnostic yield (*p* < 0.001), whereas a “moveable” SPL was not significant (*p* = 0.052). The size of the lesion was not significantly associated with increased diagnostic yield (*p* = 0.40).

**Table 1:** Baseline characteristics of patients in the study

	Ultrathin bronchoscopy (*n* = 20)	Standard size bronchoscopy (*n* = 20)	*P* value
Age, mean (SD), y	54.8 (12.4)	55.7 (12.7)	0.72
Male gender, No. (%)	10 (50.0)	8 (40.0)	0.75
Previous tuberculosis, No. (%)	4 (20.0)	4 (20.0)	0.99
Lobar localisation			0.65
Upper lobe, No. (%)	12 (60.0)	11 (55.0)	
Middle lobe, No. (%)	2 (10.0)	1 (5.0)	
Lower lobe, No. (%)	6 (30.0)	8 (40.0)	
Lesion size, median (IQR), cm	2.7 (1.7–3.5)	2.4 (1.8–3.9)	0.40
Lesion size ≥ 2 cm, No. (%)	14 (70.0)	14 (70.0)	0.99
NSCLC, No. (%)	8 (40.0)	8 (40.0)	
Benign final diagnosis, No. (%)	12 (60.0)	12 (60.0)	0.66
Tuberculosis	5 (25.0)	6 (30.0)	
Otherwise benign lesion^a^	7 (35.0)	6 (30.0)	
Confirmation of final diagnosis			0.62
Surgery	11 (55.0)	8 (40.0)	
Radiological follow-up (no histological confirmation)	8 (40.0)	11 (55.0)	
Loss of follow-up (no histological confirmation)	1 (5.0)	1 (5.0)	

^a^scar (*n* = 1), bacterial infection (*n* = 2), silicosis (*n* = 1), hamartoma (*n* = 1), unknown (*n* = 8)

*SD* standard deviation, *IQR* interquartile range, *NSCLC* non-small cell lung cancer

**Table 2:** Procedural details

	Ultrathin bronchoscopy (*n* = 20)	Standard size bronchoscopy (*n* = 20)	P value
Biopsy method, No. (%)			
Forceps	14 (70.0)	10 (50.0)	0.33
Brushing	14 (70.0)	18 (90.0)	0.23
Washing	20 (100)	19 (95.0)	0.99
Combination of all three methods	12 (60.0)	7 (35.0)	1
Combination of two methods	4 (20.0)	12 (60.0)	0.99
Washing only	4 (20.0)	1 (5.0)	1
Lesion reached, No. (%)^a^	12 (60.0)	13 (65.0)	0.99
Lesion moved, No. (%)^a^	4 (20.0)	7 (35.0)	0.48

^a^fluoroscopically determined

**Table 3:** Overall diagnostic yield and sensitivity for the diagnosis of malignancy of ultrathin versus standard size bronchoscopy

	Ultrathin bronchoscopy (*n* = 20)	Standard size bronchoscopy (*n* = 20)	*P*-value
Overall diagnostic yield	55.0	80.0	0.18
Sensitivity	50.0 (16.0–84.0)	62.5 (24.7–91.0)	0.95

Data in the table are mean values, displayed in % (95 % CI)

## Discussion

This randomised trial investigating endoscopically nonvisible SPL up to 6 cm diameter by SB showed no diagnostic benefit of switching to UB for the diagnostic bronchoscopic sampling when compared to the procedure performed with the SB alone, when used under fluoroscopic guidance. There is only limited evidence from randomised controlled trials comparing the diagnostic yield of UB with that of SB. We found that UB had no diagnostic advantage compared to SB but was more cumbersome for operators. Moreover, the procedure time was nearly five minutes longer with UB, although the difference was not statistically significant. The clinical relevance of four minutes time difference can maybe neglected, since the change from the standard to the ultrathin bronchoscope is omitted in daily routine but was part of procedure time here. Although the classical definition of SPLs considers lesions ≤ 3 cm, we have chosen to investigate lesions ≤ 6 cm, since tuberculomas of nearly double that size have been reported [10].

Data concerning the diagnostic yield of fluoroscopic guided UB compared to SB in the literature is scarce, especially for the evaluation of SPL. Considering the existing evidence in this field, the diagnostic yield of fluoroscopic guided UB in our study (55.0 %) was within the published range from 30.0 to 69.0 % reported by others [11–13], although these studies used different sampling methods and were in different populations. In a small prospective, single-arm study published by Rooney et al. on 17 patients, the diagnostic yield of UB was only 30.0 %, perhaps because only a small biopsy brush was used for the ultrathin bronchoscope [11]. The combined use of histological and cytological biopsy techniques has led to an improved diagnostic yield obtained with UB of 60.0 % [12]. Using a slightly thicker bronchoscope (outer diameter 3.5 mm) in a prospective study on 102 patients, Oki et al. reported a diagnostic yield of 69.0 % for the thin bronchoscopy irrespective of lesion size. This result was obtained without using a brush; only forceps biopsy and bronchial washing were performed [13]. We used forceps, brushing, and bronchial washings under fluoroscopic guidance as sampling methods. We did not investigate the diagnostic utility of the combined use of UB and SB in this context as sampling was only performed with the allocated bronchoscope (UB or SB). The diagnostic yield in our study may have been influenced by the prevalence of TB in our cohort (27.5 %), which is clearly higher than in the three other studies ranging from 5.9 to 12.5 %.

Technological advances in recent years have led to the availability of newer modalities helping to navigate to SPLs [14]. The weighted diagnostic yield of ENB, VB, and R-EBUS for the work-up of SPLs without the use of UB is in the range of 67–74 % which is comparable with conventional methods mentioned above [5, 15]. However, in the context of the new navigational bronchoscopic techniques the utility of UB needs to be reinvestigated. The usefulness of UB may play an important role when it is used with other new modalities such as CT-fluoroscopy or virtual bronchoscopic navigation. The combination of UB and CT-fluoroscopy is reported to have a similar diagnostic yield of 78 % in one study [16]. Data regarding VB combined with UB are conflicting. In the randomised study by Asano et al, there was no significant difference of the diagnostic yield between the VB-assisted group (67 %) and the non-VB-assisted group (60 %) [17]. However, under the combined use of EBUS, fluoroscopy, and virtual bronchoscopic navigation guidance, a recent randomised study published by Oki et al. could show that the diagnostic yield for malignant lesions was significantly higher with a 3.0-mm ultrathin bronchoscope (74 %) compared to a 4.0-mm thin bronchoscope (59 %) [18]. The authors concluded that the combination of UB with navigational technology and endobronchial ultrasound (EBUS) seems to combine the best two modalities for evaluating peripheral pulmonary lesions [18]. Interestingly, the diagnostic yield of UB was markedly higher in the study by Oki et al. (74 %) compared to our study (55 %). In contrast, the yield of SB was higher in our study (80 %) compared to 59 % by Oki et al., although a slightly thinner bronchoscope was used in their study [18]. On one hand, this underlines the conclusion that advanced navigational technologies improve the diagnostic yield of UB. On the other hand, these differences raise the issue of the operator’s experience and a possible influence by the higher TB prevalence in our cohort. An additional aspect to consider is that VB- or ENB-guided biopsy techniques increase average costs, which is not feasible in many parts of the world [19]. Compared to video-assisted thoracoscopic surgery (VATS) or (18)F-fluoro-deoxyglucose positron emission tomography (FDG-PET), ENB is more cost-effective to diagnose lung cancer in moderate- to high-risk patients [20]. Another issue, which should be considered, is training. The recommended training requirement for R-EBUS is at least 50 supervised procedures [21]. Issues of costs and training need to be considered before acquisition and introduction of such modalities. Some of these diagnostic modalities may not be feasible in less affluent settings. In addition, the benefit of these highly sophisticated diagnostic tools in an area with high incidence of TB remains questionable.

The limitations of this study are its small sample size, since it was originally designed as pilot trial, and that not all bronchoscopic results were confirmed by surgical sampling. Interestingly, none of the individuals in whom observation was considered the appropriate strategy by the clinical board had a malignant lesion. Furthermore, a new ultrathin bronchoscope with a larger working channel has been introduced which most probably leads to higher yields [13]. All of the UB procedures were done with a fiberscope, which might have influenced diagnostic yield and could have caused a bias. We did not evaluate the distance of SPL from hilum or presence of bronchus sign as possible predictors of the diagnostic yield. Lastly, the bronchoscopies were performed by five different operators, which may have introduce a certain bias. However, three of the five operators had a documented track record from a previous study, and at least one of them was present in the bronchoscopy suite for all study related procedures [22].

## Conclusions

We conclude that the inspection and sampling with UB in endoscopically non-visible SPL showed no advantage over sampling with SB in the evaluation of undiagnosed SPL in a TB-endemic area, when combined with fluoroscopic guidance only. It seems that for the diagnostic work-up of SPL, tumour visibility is less important than getting higher volumes of material. Sample material volume consisting of tumour cells and fluid markers is determined by the diameter of the bronchoscope.

## Ethics approval and consent to participate

Written informed consent was obtained from all patients before inclusion in the study, which was approved by the Committee for Human Research of Stellenbosch University (Cape Town, South Africa) with the reference number 2000/C094. The study is registered at ClinicalTrials.gov (Identifier: NCT02490059).

## Availability of data and materials

The dataset is deposited publicly as additional supporting file.

## Additional file

Additional file 1: The supplemental file. (XLS 16 kb)

## Abbreviations

AE: adverse event
CT: computed tomography
ENB: electromagnetic navigation bronchoscopy
IQR: interquartile range
R-EBUS: radial endobronchial ultrasound
SB: standard size bronchoscopy
SD: standard deviation
SPL: solitary pulmonary lesion
TB: tuberculosis
TBB: transbronchial biopsy
UB: ultrathin bronchoscopy
